# The external quality assessment scheme: Five years experience as a participating laboratory

**DOI:** 10.4103/0973-6247.59388

**Published:** 2010-01

**Authors:** Rajendra Chaudhary, Sudipta S. Das, Shashank Ojha, Dheeraj Khetan, Atul Sonker

**Affiliations:** *Department of Transfusion Medicine, Sanjay Gandhi Postgraduate Institute of Medical Sciences, Lucknow - 226 014, India.*

**Keywords:** External quality assessment scheme, quality control, quality assurance

## Abstract

**Background and Aim:**

Quality assurance in blood banking includes active participation in the external quality program. Such a program offers valuable benefits to patient care, their safety, and an overall quality of laboratory practices. In the year 2002, we participated in the External Quality Assessment Scheme (EQAS) under the World Health Organization (WHO), Bureau of Laboratory Quality Standards, Thailand.

**Materials and Methods:**

In the current study we evaluated our EQAS test result of the past five years, from 2003 to 2007. Test results of all blood samples such as ABO grouping, D typing, antibody screening, antibody identification, and transfusion transmitted infection (TTI) testing were analyzed and documented.

**Results:**

Discordant results in one or more instances were observed with antibody identification, weak D testing, and tests for anti-HIV1/2 and HBsAg. Twice we failed to detect the ‘anti-Mia’ antibody in the issued sample and that could be attributed to the absence of the corresponding antigen in the used cell panel. HBsAg was missed due to its critically low titer in the serum and the comparatively low sensitivity of our Enzyme-Linked Immunosorbent Assay (ELISA) test kit.

**Conclusion:**

All these failures in the last five years have helped us to significantly improve our transfusion service in terms of performance evaluation, patient care and safety issues, and the overall quality of laboratory practices. We therefore recommend all laboratories and hospitals to participate in the EQAS program, which will definitely help them to improve from what they learn.

## Introduction

Quality system essentials (QSE), the guiding principles to any laboratory quality program, were established by the Clinical Laboratory Standards Institute (CLSI) in 1991.[1] As many as ten or more major laboratory activities were developed under these essentials to ensure accurate laboratory practices that would serve the needs of patients as well as clinicians. Quality control and assurance is an important component of QSE that not only helps to carry out a laboratory quality program systematically, but also dictates active participation in external quality assessment (EQA) or proficiency testing (PT).[2] Such a program offers valuable benefits to the participating laboratory in terms of performance evaluation, improvement in patient care and safety, and the overall quality of laboratory practices.[3–7] The organizing laboratory that conducts such an EQAS periodically assesses the registered participating laboratory. Such a registration is not mandatory, but absolutely voluntary. To review and assess the quality of laboratory and clinical practices, our transfusion service was registered in mid 2002, under the Bureau of Laboratory Quality Standards, Department of Medical Sciences, Ministry of Public Health, Thailand. This EQA program was termed as the Regional EQAS (REQAS), which included countries under the South-East Asia Regional Office (SEARO) of WHO. Since 2003, we as a participating laboratory under REQAS, Thailand, have been receiving blood samples, thrice a year, for serological and immunohematological testing. We present here our experience with EQAS for the past five years.

## Materials and Methods

### Blood samples

EQAS blood samples from the Bureau of Laboratory Quality Standards, Department of Medical Sciences, Thailand, were received and processed at the Department of Transfusion Medicine at our Institute.

For each year (2003-2007), every four months, samples were shipped to our center for specific tests recommended by the organizing laboratory, following the IATA guidelines for shipment of biological materials. Each time, unknown samples consisting of red cell suspension or serum or both, all packed with coolant were received within three days of dispatch. All the samples were handled as part of routine work samples and recommended tests were performed by the concerned laboratory technician on duty. The tests were performed on the same day of receipt of the samples and results mailed to the organizing laboratory within a week.

### Test performed on blood samples

Every year, a total of 27 blood samples (nine samples every four months) for red cell serology and 24 sera (eight samples every four months) for TTI were received. The serological tests recommended by the organizing laboratory included ABO grouping, D typing including Du, antibody screening, and antibody identification. Tests for TTI included HBsAg and anti-HIV antibody testing. All tests were performed by dedicated staff using the conventional technique available in the department. Tests for HIV-1, HIV-2, and Hepatitis-B were performed by the routine conventional sandwich ELISA. ABO grouping and D typing, including D_u_ testing, were performed by the conventional tube technique (CTT), following the standard operating procedure (SOP) of the department. Antibody screening and identification were done using CTT till November 2004, and later on by gel technology (GT), using commercial panels.

## Result

Table 1 depicts the EQAS test result for red cell serology in the last five years, from 2003 to 2007. While a 100% concordance was observed in ABO grouping and antibody screening, in one instance a ‘weak RhD’ antigen was missed (concordance 89%). The Anit-Mia antibody could not be identified on two occasions.

**Table 1:** External quality assessment scheme test result: Red cell serology (2003-2007)

Year	Total samples received (N)	ABO group		D typing		Antibody screen		Antibody identify	
					
		CR (%)	DR (%)	CR (%)	DR (%)	CR (%)	DR (%)	CR (%)	DR (%)
2003	18	100	0	100	0	100	0	NP	NP
2004	27	100	0	89	11	100	0	100	0
2005	27	100	0	100	0	100	0	100	0
2006	27	100	0	100	0	100	0	100	0
2007	27	100	0	100	0	100	0	77.8	22.2

CR - Concordant result; DR - Discordant result; NP - Not participated

A discordant result was observed in two sample sets, one each for HIV and HBsAg testing [Table 2]. In the first set an HIV non-reactive sample was reported as a ‘gray zone’ sample (concordance 93.8%) and in the other set, serum HBsAg could not be detected. In both these instances the EQAS result was low scoring and labeled as ‘unsatisfactory’.

**Table 2:** External quality assessment scheme result: Transfusion transmitted infection testing (2003-2007)

Year	HIV ½			HBsAg		
		
	No. of samples (N)	CR (%)	DR (%)	No. of sample (N)	CR (%)	DR (%)
2003	16	93.8	6.2	3	100	0
2004	24	100	0	2	50	50
2005	24	100	0	NR	-	-
2006	24	100	0	NR	-	-
2007	24	100	0	NR	-	-

CR - Concordant result; DR - Discordant result; NR - Sample not received

## Discussion

The EQAS program is a valuable management tool destined to improve the efficiency and service of a laboratory in particular and a hospital in general. The program provides an opportunity to the participating organizations to compare activities and modify their own practices based on what they learn.[2,7] In a transfusion service, EQAS evaluates the performance of procedures, equipment, materials, and personnel, and suggests areas for improvement.[7,8]

As a participant of the REQAS, we performed all the prescribed tests by strictly following the departmental SOP and manufacturer's instruction, considering each lot as routine working samples. In all instances, we could perform the ABO grouping that comprised of both the cell and serum grouping accurately. We unfortunately failed to detect a red cell sample of a ‘weak D’ phenotype in the last lot of 2004. This could be attributed to the failure of the monoclonal anti-D (blend of IgG + IgM) to detect weak D.

Although screening of the antibody was never a problem with our EQAS testing, twice in the recent past we failed to identify the implicated antibody in the serum sample. In both these instances, the designated antibody was ‘Anti-Mia’ and failure could have been due to the absence of the corresponding antigen (Mia) in the available commercial antibody identification 11 cell panels (DiaMed, Switzerland). As a result of this, we currently use antibody detection panel containing Mia antigens so that no sample with the ‘Anti-Mia’ antibody is missed [Asia (Mi^a^ +), DiaMed, Switzerland]. ‘Anti-Mia’ antibodies react with the Mi III phenotype and the frequency of this phenotype, of the Miltenberger subsystem, varies from region to region. Although it is rarer in the western population, its frequency is relatively common in Southeast Asia, especially along the south-east coast lines of China and Taiwan. In one study performed on the multiethnic Peninsula's Malaysian population, consisting of 33,716 Chinese, Malay, and Indian patients, the Mia phenotype prevalence was estimated to be 0.3, 0.2, and 0.2% in Chinese, Malay, and Indian subjects, respectively.[9] In another Chinese series the Mi-III frequency was observed to be 4.7%, almost 500 times higher than in Whites.[10] India being a vast country comprising of multiethnic population, the Mi phenotype might vary from state to state. Therefore, such a phenotype study is now essential on a regional basis, so that a blood transfusion service may prepare its own in-house red cell panel for antibody screening and identification.

In each lot we received eight serum samples of varying dilutions for determination of anti-HIV1/2 antibodies. In one instance in 2003, one of the duplicate samples was labeled in the ‘gray zone’ for HIV, although originally it was a non-reactive serum. Subsequently a technical error with the ELISA reader was confirmed and the problem was resolved immediately in collaboration with the manufacturer. This highlights the importance of a periodic quality control of all equipments and reagents in a laboratory. Such a practice not only furnishes the outcome of a correct result, but also maintains confidence in the facility. In another instance we failed to detect an HBsAg infected serum sample. Here the serum dilution was so critical that our ELISA system failed to detect the antigen and labeled the serum as non-reactive. This reflected low sensitivity of the test kit used and immediately the problem was reviewed and solved through procurement of fresh ELISA kits of a different manufacturer, and these kits were brought into routine laboratory use only after establishing their quality.

## Conclusion

An EQAS program plays an important role in improving the efficiency of a laboratory service, thereby optimizing the overall quality of a health care system. In the last five years we could significantly improve our transfusion service in terms of performance evaluation, patient care and safety issues, and overall quality of laboratory practices. We believe that global participation in such an EQAS program will definitely improve the quality of a hospital service because no health care facility can be totally self-sufficient and there is always an inclination for improvement and development in a system.
